# System-level barriers account for non-compliance to physiotherapy among persons with Parkinson's disease at the Korle Bu Teaching Hospital, Ghana: an explanatory mixed-method study

**DOI:** 10.4314/ahs.v24i4.42

**Published:** 2024-12

**Authors:** Mary W Agoriwo, Pascal G Adorvlo, Peter Oppong Junior, Ellen Mensa-Bonsu, Martin Ackah, Benedicta Atsivor

**Affiliations:** 1 University of Health and Allied Sciences, Department of Physiotherapy and Rehabilitation Sciences; 2 Korle Bu Teaching Hospital, Department of Physiotherapy

**Keywords:** Parkinson's disease, physiotherapy, patient compliance

## Abstract

**Background:**

Despite the increasing importance of physiotherapy and robust evidence, there is still limited studies assessing compliance rates and barriers to physiotherapy appointments for Persons with Parkinson disease (PwPD) especially in low-resource settings such as Ghana.

**Objective:**

To assess compliance rates and identify barriers to adhering to physiotherapy appointments among PwPD receiving physiotherapy management at a tertiary hospital in Ghana.

**Methods:**

A sequential explanatory mixed method design was used. The quantitative part involved a retrospective survey of health records of PwPD who reported at the Korle-Bu teaching hospital physiotherapy PD-clinic from 2013-2021. The qualitative part involved semi-structured telephone interviews among defaulters from the year with highest clinic attendance. Quantitative data was descriptively analyzed. For the qualitative data, a deductive qualitative content analysis of the transcribed audiotaped interviews was conducted.

**Results:**

Eighty-six PD records (56 males) were included. Overall mean (SD) age was 67(±11) and ranged from 38-90 years. The year 2014 recorded the highest number of attendees (n=20/86; 23%). Overall, 88% of the participants had stopped physiotherapy. Six PwPD participated in the interviews. System-level barriers were mainly recorded as the reasons for patients defaulting physiotherapy.

**Conclusion:**

The majority of PwPD had stopped physiotherapy and system-level barriers were the key reasons.

## Introduction

Although there is no cure for Parkinson Disease (PD), physiotherapy is essential in enhancing the quality of life of persons with PD (PwPD) and promoting their social integration into the community^1^. Clinically, starting an exercise program and complying with it has been shown to improve self-reported health-related quality of life and mobility^2^. It is reported that disease progression can be modified through exercise as the brain can undergo neuroplasticity with increased corticomotor excitability and enhanced connectivity between the diseased basal ganglia and the brain's cortex^3^-^5^. Additionally, exercise helps reduce global brain atrophy and improves cognitive function^6^. PD is a progressive neurodegenerative disease characterized by both motor (resting tremors, bradykinesia, rigidity, and postural instability) and non-motor (autonomic dysfunction, sleep disturbances, mood disorders, and other neuropsychiatric disorders) features^7^,^8^. The occurrence of motor and non-motor symptoms is linked to the loss of striatal dopaminergic neurons^9^,^10^. As a result, the inhibitory impact of the basal ganglia on other brain areas involved in the regulation and execution of voluntary movements (such as the thalamus, brainstem, and supplementary motor area) becomes amplified, leading to bradykinesia and rigidity^11^. Balance problems, on the other hand, may be caused by problems with the non-dopaminergic system, which is typically resistant to dopamine^12^. These neuronal changes result in functional problems which affect the daily activities of living among PwPD resulting in decreased quality of life. Although exercise does not stop PD from advancing, it reduces the rate of progression of the disease, improve function and quality of life of the affected persons^2^. Generally, physiotherapy has been found to improve motor symptoms, gait, balance, and quality of life of PwPD^2^. The physiotherapy interventions are in the form of conventional physiotherapy (aerobic exercises, overground walking, strengthening, postural correction by wall-bar, staircase climbing etc.), balance and gait training, dance, Nordic walking, hydrotherapy, treadmill walking, dual task training, martial arts, and exergaming (Radder et al 2020). To achieve significant benefit from physiotherapy, adherence to prescribed interventions especially, exercises is paramount. Zaman, Ghahari & McColl^13^ reported that PwPD who adhere to consistent regular exercises yield substantial improvements in functional balance and mobility irrespective of exercise intensity as compared to those who are non-adherent. However, most PwPD often remain sedentary in everyday life, and achieving good adherence to an active lifestyle remains a great challenge. Additionally, factors such as cost of treatment and transportation, waiting time, and lack of equipment among others have been cited as causes of non-adherence to healthcare^13^-^15^. These barriers could have an impact on the rate of compliance with physiotherapy management^1^. Hence, identifying context-specific barriers to utilizing physiotherapy among PwPD in Ghana is imperative to inform measures to reduce non-compliance. In Ghana, studies conducted at the adult neurology clinics of two teaching hospitals recorded PD as the top hypokinetic disorder^16^, and among the three most common non-communicable diseases, after stroke and epilepsy, increasing the burden of neurological disorders^17^. Given the growing significance of exercise/physiotherapy and strong evidence supporting it coupled with the limited research on physiotherapy compliance, this study aimed to evaluate the rate of compliance and identify barriers to adhering to physiotherapy appointments among PwPD undergoing physiotherapy management at a tertiary hospital in Ghana. The study's specific objectives were to: a) determine the number of PwPD who reported to the physiotherapy PD-clinic; b) determine the compliance rate of physiotherapy attendance among the PwPD; and c) establish the barriers for non-adherence to physiotherapy appointments, within the period under review (May 2013 - December 2021).

## Material and methods

### Study site and design

The study was conducted at the PD clinic of the physiotherapy department of the Korle Bu Teaching Hospital (KBTH), Ghana. This PD clinic was established in May 2013 to provide PD-specific physiotherapy services to PwPD. A sequential explanatory mixed-method design was employed involving a quantitative retrospective survey of patients' health records followed by semi-structured in-depth telephone interviews.

### Study population and sampling

In the quantitative study, only medical records of patients with confirmed diagnosis of PD by a neurologist were included. For the qualitative study, PwPD who had defaulted treatment in the year with the highest number of attendees (2014, n=20), per the medical records retrieved, were purposefully selected. This was to take care of the anticipated likelihood of not reaching some of the patients and to ensure that the interview participants shared similar experiences with physiotherapy as much as possible. According to Chan et al., (18), a defaulter is defined as a person who refuses, delays or discontinues treatment or who did not attend three or more consecutive visits after their last clinical appointment. Within a year, patients are expected to accumulate a maximum of 16 sessions of physiotherapy. Thus, weekly physiotherapy sessions for four weeks, followed by two sessions fortnightly, and monthly for the remaining ten months. Therefore, in this study, a defaulter was defined as a PwPD who did not attend at least three consecutive visits after their last physiotherapy session within 12 months of physiotherapy. Hence a PwPD who defaults physiotherapy treatment was described as being non-compliant.

### Data collection instrument

A data collection tool (Appendix 1) was developed to retrieve relevant information, such as patients' demographic details, telephone numbers, first date of attendance, date of last attendance, and current state of physiotherapy attendance from the medical records of PwPD.

A semi-structured interview guide (Appendix 2) which focused on asking questions that prompts the respondent to give reasons for defaulting physiotherapy sessions was designed for the telephone interview. The questions in the interview guide were related to system-level barriers and individual-level barriers as the two types of barriers to healthcare access^13^. Under the system-level barriers, questions focused on cost of treatment and transportation, availability of equipment, treatment duration and waiting time. Questions on the individual-level barriers focused on treatment satisfaction and expectations, family and social support, and comorbidities. The data collection form and interview guides were both piloted and revised prior to their use to ensure that no relevant information was left out. The form was piloted with nine randomly selected medical records from the included records. These records were included in the study. The interview guide was piloted with a female and male PwPD aged 56 and 65 years, respectively. Wording of questions and approach to questioning were revised accordingly.

### Data collection process

Medical records of patients who attended the PD-clinic from 2013 to 2021 were retrieved with the assistance of a records manager. Eligible medical records were selected, and relevant data extracted using the data extraction form for the quantitative study. For the qualitative aspect of the study, defaulted patients in the 2014 year were contacted via telephone to discuss their availability and willingness to participate in the interview after the objectives of the study had been explained. Appropriate times for the interviews were scheduled for those who expressed interest to participate. Treatment defaulters who could not be reached via telephone after three consecutive calls on three consecutive days at different times of the day were excluded from the study. For patients with cognitive or speech impairments, their caregivers were interviewed. One of the researchers' conducted the telephone interviews. After the purpose of the study was explained, a verbal consent was sought to conduct and record the interview. Upon reaching the participant on telephone at an agreed scheduled time, the researcher did an introduction, explained the study objectives, and asked permission to conduct and record the interview. The researcher asked each question on the interview guide and probed wen needed. After the participant had responded appropriately to all the questions, the researcher thanked the participant and ended the telephone call. Each interview lasted for about 20 minutes. Ethical approval was obtained from the University of Health and Allied Sciences Research Ethics Committee (UHAS-REC A. 10[5]21-22) and the KBTH Ethics Committee (KBTH-ADM/00095/2022).

### Data analysis

The data was collated using Microsoft Office Excel 2016. Descriptive statistical analysis was conducted for the quantitative data using Statistical Package for the Social Sciences (SPSS), v26.0. Frequencies, percentages, means, standard deviations, and ranges were calculated and illustrated in tables and charts. For annual compliance rate, the actual number of physiotherapy PD-clinic attendance was divided by the expected number of attendance and multiplied by 100. The audiotaped interviews were transcribed verbatim for qualitative analysis. Qualitative content analysis was conducted for the transcribed data. Microsoft Excel (2016) was used to input, clean and categorize transcribed data under two themes and seven sub-themes set a priori after transcripts were read several times. Umbrella terms for system-related and patient-related factors that reflect the types of barriers to healthcare access were formulated as system-level barriers and individual-level barriers respectively, as themes. Four sub-themes, inaccessibility and cost of transportation, cost of treatment, treatment duration and waiting time, and non-availability of equipment, were created under the system-level barriers theme. The individual-level barriers were also further grouped under three sub-themes, dissatisfaction and unrealistic expectation of therapy, presence of comorbidities, and family and societal support. This deductive approach to the qualitative data analysis was relevant to enable the researchers to apply the pre-determined themes and sub-themes derived from existing literature to the data^19^,^20^.

## Results

### Socio-demographic characteristics of patients

A total of 86 medical records of PwPD (56 men: 65%) who visited the PD clinic met the inclusion criteria out of 94 records retrieved from May 2013 to December 2021. This resulted in a male-to-female ratio of approximately 2:1. The mean (SD) age was recorded as 67(±11) years and ranged from 38-90 years old. The year 2014 recorded the highest number of patients (n=20/86; 23%) with 2021 recording the least (n=3/86; 4%). The majority of the patients were retired (n=53/86; 62%) with a few unemployed (n=3/86; 4%). Table 1 presents further details of the demographic characteristics and number of incident cases per year (May 2013 - December 2021).

**Table 1:** Socio-demographic characteristics of patients with Parkinson's disease

Year	Number of patients	Age range	Mean Age (SD)	Gender ratios (M:F)	Profession				
					G.W(%)	P.W(%)	S.E(%)	R.T(%)	U.E(%)
**2013**	11	51-83	68(9.5)	10:1	1(9)	2(18)	1(9)	7(64)	-
**2014**	20	46-76	62(9.1)	10:10	-	3(15)	3(15)	12(60)	2(10)
**2015**	14	38-87	67(14)	9:5	1(7)	1(7)	1(7)	10(71)	1(7)
**2016**	6	57-78	69(9.5)	4:2	1(17)	-	1(17)	4(67)	-
**2017**	9	53-90	75(12)	7:2	-	1(11)	2(22)	6(67)	-
**2018**	4	51-81	69(14)	4:0	-	-	2(50)	2(50)	-
**2019**	6	38-83	64(17)	5:1	1(17)	-	3(50)	2(33)	-
**2020**	13	51-80	66(8.9)	6:7	-	3(23)	3(23)	7(54)	-
**2021**	3	66-79	74(6.8)	1:2	-	-	-	3(100)	-

**2013-2021**	86	38-90	67(11)	56:30	4(5)	10(12)	16(19)	53(62)	3(4)

***Key: SD = Standard Deviation, M:F = male: female, G.W = government workers, P.W =private workers, S.E = self-employed, R.T= retired, U.E = unemployed***

### Compliance rate and current state of physiotherapy

The overall average rate of compliance for physiotherapy of the 86 PwPD within the years under review was 67%. The year with the highest compliance rate was 2019 (100%) while 2016 recorded the lowest compliance rate, 41% (table 2). However, the majority of the PwPD (n=76/86; 88%) had defaulted and stopped attending the physiotherapy clinic. Only 5 (6%) PwPD were still attending the clinic and 5 (6%) patients had died within the period under review (Figure 1).

**Table 2:** Expected and actual average visits with annual compliance rate

Years	Average of expected visit (range)	Average of actual visit (range)	Annual rate of compliance (%)
**2013**	24.2 (2-59)	10.2 (2-24)	42
**2014**	12.0 (1-42)	6.0 (1-18)	50
**2015**	10.4 (1-37)	5.6 (1-18)	54
**2016**	14.5 (1-39)	6.0 (1-22)	41*
**2017**	12.2 (1-62)	8.8 (1-48)	72
**2018**	10.0 (4-22)	8.8 (2-22)	88
**2019**	11.4 (8-15)	11.4 (8-15)	100**
**2020**	12.4 (3-24)	10.6 (2-24)	85
**2021**	13.0 (12-14)	12.7 (12-14)	97

**2013-2021**	13.3	8.9	**67#**

** *Highest compliance rate*

* *Lowest compliance rate*

# *Overall compliance rate*

**Figure 1:**
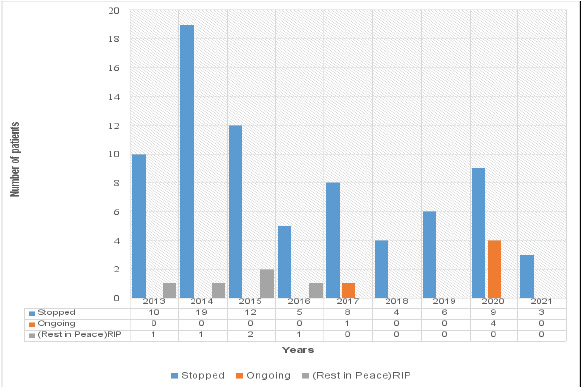
Current state of physiotherapy clinic attendance of the patients

## Qualitative findings

### Socio-demographic characteristics of interview participants

Out of the 19 defaulted PwPD for 2014 contacted via telephone calls, 6 (32%) were willing to participate in the interview, 2 (11%) declined and 11 (58%) could not be reached. The three males and three females interviewed had a mean (SD) age of 65 (±8.1) and ranged from 52-74 years. Participants travelled a mean (SD) distance of 19 (±9.3) Km to access physiotherapy. All participants were married and lived with their family (Table 3).

**Table 3:** Socio-demographic characteristics of patients with Parkinson's disease

Participant	Age (Years)	Gender	Occupation	Distance travelled, Km
P01	61	Male	Retired Banker	15.0
P02	52	Female	Trader	24.0
P03	69	Male	Pensioner	4.0
P04	62	Male	Retired	29.0
P05	71	Female	Retired	29.0
P06	74	Female	Trader	12.0

**Average distance covered (SD)**				**19.0 (9.3)**

*SD=Standard deviation*

### Overview of major themes

In order to promote the ‘voices’ of the participants, each sub-theme is illustrated and supported by an example of a significant participant statement(s). Some patients had assistance from their caregivers in answering some of the questions, however, such responses were considered as one patient ‘voice’.

#### Theme 1: System-level barriers

##### Sub-theme 1: In accessibility and cost of transportation

Accessibility to the facility and the cost involved in transportation were reasons for defaulting physiotherapy treatment as mentioned by the participants. Half of the participants' (n=3/6; 50%) means of transportation was via taxi (P02, P05, and P06) and the rest (n=3/6; 50%) came with their own vehicles. On average it cost about GHȻ100.00 ($10.00) to get to the physiotherapy clinic, and return home, which was generally considered expensive. On average, patients spent about 45minutes or an hour to get to the physiotherapy clinic which most of the participants (n=5/6; 83%) indicated was a fator for stopping physiotherapy. Relevant quotations for the subsequent themes and sub-themes are detailed on table 4.

**Table 4:** Relevant quotations to support themes and sub-themes

Themes and sub-themes	Quotations
**Theme 1: System level barriers**	

Sub-theme 1: inaccessibility and cost of transportation	**Quotes for cost of transport**
	(P02) *“I take ‘dropping’ and it was GHȻ 50.00. It was too expensive, too much”*.
	P03 *“I buy fuel around GHȻ 100.00 but we don't spend all the GHȻ 100 there. I will say its moderate by then but now I will say it's expensive”*.
	P05; *“The distance, we are now close to Kokrobite and coming down to KBTH for physio was becoming a problem. It cost GHȻ 100.00 that's GHȻ 50.00 in and GHȻ 50.00 out. It's too expensive, ehh I am a retiree”*.
	**Quotes for travel duration**
	P01 reported *“So, we were coming with a taxi, from Ofankor to Korle Bu and it was quite a distance and without traffic it should take 40mins but with traffic an hour”*.
	P02 responded “*like one hour*” “*Err, at times we leave home around 7:00 or 7:30 am and we get to Korle Bu around 8:30am or 9:00am, It is quite far”*.
	P05 stated *“Because of traffic it takes about an hour and half”*. *“It was becoming tiring for me (the husband) and herself if I had a physio nearby, I would have love it so we started going to Akawe*, *that was a reason she defaulted”*.

Sub-theme 2: Cost of treatment	P03 stated *“Ohhh, wow because first I have to use the national health insurance and may be some little money like* GHȻ *20.00 by then but it was under* GHȻ *50.00” and “the cost was worrying, that everyday every time I come, I have to pay money why? I use to complain about it. Because every session I have to pay money. Ohhhh it was moderately expensive”*.
	P05 said *“we paid about GHȻ 100.00 during visit to the physiotherapy clinic and it was expensive but this was not the reason why she defaulted physiotherapy treatment”*.

Sub-theme 3: Treatment duration and waiting time	**Quotes for treatment duration**
	P01 said *“treatment time was about 45mins which was okay”*.
	*P06 said “Treatment took about 30 to 40 mins and it was okay”*.
	*P03 said “And the treatment time was under 40 minutes, it somehow too little, ermm, because sometimes they will be like we are teaching you so that when you go home you can do it there”*.
	**Quotes for waiting time**
	P01 said *“I had to wait for about 30 mins and that was okay”*.
	P04 said, *“Usually not long, they are booked so if they book you for 10 O'clock usually, they see you when you get there, the waiting time is not that long it was about 30-40mins”*.
	P06 said, *“if I get there early, I can wait for about 15mins before they see me and if I get there late, I meet a lot of people so I have to wait for about 40mins before they see me ”*.

Sub-theme 4: Non-availability of equipment	P02 reported *“there was no equipment and no access to all equipment”*.
	P05 noted that *“we had to wait until those people who were there to finish. So, I think unavailability of sufficient equipment made me stop the physio”*.

**Theme 2: Individual level barriers**	P02 reported “*I was not satisfied with treatment*”.
Sub-theme 1: Dissatisfaction and unrealistic expectation of therapy	P03 said *“my expectation was like as I start the physiotherapy I recover soon and since that expectation wasn't met that prevented me from attending. Ermm, the satisfaction, I thought I will get a quick response to healing and since I did not get it that is why I stopped. I wasn't much satisfied”*.
	P06 reported *“I am satisfied, but I was not seeing much improvement because every day same thing and home assignment but I am not improving very fast. I expected to get better as soon as possible. I will say my expectations were not met so I stopped”*.

Sub-theme 2: Presence of comorbidities	P04 said, *“I am diabetic but it is not the reason why I stopped physiotherapy”*
	P05 said, *“Yes, she is diabetic and hypertensive. The hypertension was alternating, so sometimes in a hurry we may forget the medication in attempt to reach the physio department early. The blood pressure will be high and she cannot be seen so we stopped”*.

Sub-theme 3: Family and societal support	**Quotes for family support**
	P01 *“it was good”*. P06 said, *“My husband and children are always there for me”*
	P02 recounted, “*the family support was poor and that made me stop physio”*.
	***Quotes for societal support***
	P02 said, “No I wasn't stigmatized”
	P03 said, “Cordial, he wasn't stigmatized just that he is not happy when he is in the mist of people and he start shaking. So, because of that he doesn't attend programs, not the society but him personally”.

##### Sub-theme 2: Cost of treatment

Participants expressed their concern on the cost of treatment as a reason for defaulting treatment. All the participants reported they had to pay money before they received physiotherapy treatment. They paid an average of about GHȻ30.00 ($3.00) with insurance and about GHȻ50.00 ($5.00) without insurance on each visit which made some of the participants, P02 and P03 (n=2/6, 33%) stop physiotherapy due to treatment cost. Although, P05 and P06 also indicated treatment cost was expensive, that was not the reason for default (table 4).

##### Sub-theme 3: Treatment duration and waiting time

Participants expressed their views about the time spent during therapy and waiting time before therapy as possible reasons for defaulting treatment. An average treatment time as reported by the participants was 30 minutes and varied from 30 minutes to an hour which was acceptable to the majority (n=5/6, 83%) of the participants except P03 who said the treatment time was too little (table 4).

Regarding waiting time, participants spent an average time of about 30 minutes of waiting before they were seen and this ranged from 15 minutes to about 1 hour. However, majority (n=5/6, 83%) of the participants did not see this as a problem or reason for default except P02 who reported of about 3-4 hours waiting which was considered too long (table 4).

##### Sub-theme 4: Non-availability of equipment

Although the participants had little knowledge on equipment needed for treatment session, 50% of the participants (n=3/6) were satisfied with equipment availability. However, 2 (33%) of the participants (P02 and P05) said that the equipment were either unavailable or in limited access for use by the patients (table 4).

#### Theme 2: Individual level barriers

##### Sub-theme 1: Dissatisfaction and unrealistic expectation of therapy

Dissatisfaction with treatment was a reason for defaulting (P02 and P03) treatment, though, others were partly satisfied (n=4/6, 67%) with the overall physiotherapy sessions. Notwithstanding, the treatment expectations of the majority (n=5/6; 83%) were not met and contributed to them defaulting physiotherapy (table 4).

##### Sub-theme 2: Presence of comorbidities

Three (50%) participants reported of having some comorbidities. P04, P05, and P06 reported hypertension and diabetes mellitus as their comorbidities. However, only P05 stopped attending physiotherapy due to the hypertension (table 4). The others reported no comorbidities.

##### Sub-theme 3: Family and societal support

Family support was not a barrier to treatment as reported by five (83%) participants: P01, P03, P04, P05 and P06 (table 4). Although, P05 reported to have family support, she defaulted due to caregiver burden, she said “We (patient and caregiver) calculated everything and we stopped, we were doing it twice a week and it was becoming too cumbersome for the old man (caregiver)”. Only P02 specifically reported poor family support as a barrier to physiotherapy (table 4). All six participants had no challenges with attitude of society towards them (table 4).

## Discussion

The study revealed that a significant number of PwPD who sought care at the physiotherapy department's PD-clinic in KBTH from May 2013 to December 2021 had defaulted their physiotherapy treatment. Moreover, the findings indicated a higher prevalence of PD among men compared to women in PwPD who reported at the physiotherapy PD-clinic within the period under review. The primary reasons for non-adherence to physiotherapy were identified as system-level barriers. Considering the significant effect of physiotherapy interventions in restoring motor and non-motor functions in PwPD, continuous utilization of physiotherapy is paramount to optimal benefit and stalling of disease complication.

On average, the PwPD whose medical records were reviewed within the nine-year period under consideration recorded a 67% rate of compliance to physiotherapy management during their treatment years. This score appears deceptive as 88% of the study participants were no longer attending physiotherapy. In a study by Seo et al., 21, there was no remarkable change in the rate of rehabilitation therapy utilization from 2004-2015 among Korean PwPD. However, the number of PwPD who repoted at the physiotherapy PD-clinic fluctuated over the study period. Since the physiotherapy PD-clinic mostly relied on referrals from the adult neurology clinic of the hospital, reduced referral of PwPD to physiotherapy by the doctors or neurologist could account for the intermittent decline observed. Additionally, the discontinuation of physiotherapy among the PwPD recorded over the period under review could be attributed to the several factors mentioned by the interview participants. These factors encompassed challenges related to problems with accessibility and transportation costs, as patients resided relatively far from the hospital, and faced significant expenses for transportation. Other obstacles included treatment expenses, limited treatment duration, dissatisfaction with treatment, perceived lack of equipment, and inadequate family support. Cost of transportation and inaccessibility to the treatment facility were factors that influenced the rate at which PwPD utilized physiotherapy. Patients used private or public means of transport and travelled 4Km to 29Km for about 45-60minutes to access physiotherapy. Although, access to transportation was not a problem, patients were concerned about the high cost involved in either paying for the public transport or fueling private vehicle (GHȻ 100.00 ($10.00)) which resulted in many defaulting. This confirms Zhou's^22^ finding that people who travel 30minutes or more to access medical care are likely to experience increased delay or skipped medical care compared to those who travel less than 15minutes. Participants wished they had other physiotherapy PD-clinic facility closer to their place of residence for easy access and reduced waiting time as they could get to the facility early and on time.

Participants with or without insurance had to pay some amount of money before accessing treatment on each visit which was burdensome. Zaman, Ghahari & McColl^13^ have reported on the financial hardships of PwPD paying out of pocket, not having health insurance, and lack of insurance coverage. Participants echoed their concerns about the financial burden of receiving the physiotherapy treatment as they had to pay on every visit which resulted in their defaulting of treatment. This shows that a complete insurance coverage could have facilitated adherence to physiotherapy for the study participants. About 84% of the participants reported on waiting time of about 30 minutes or more before they were seen which was not a reason for defaulting physiotherapy treatment. in contrast to Mohiuddin's^14^ study prolong waiting time was one of the key barriers to accessing healthcare in Bangladesh. Although participants were seen according to appointments, the large number of patients reporting at the department could account for some delays. As confirmed by one of the participants, a patient is seen promptly when there are few of them at a time. The participants spent about 30-60 minutes of exercise sessions on each visit and this complies with the American College of Sports Medicine (ACSM) recommended duration for exercise program^23^. Home exercise programs were also prescribed though, details of participants specific physiotherapy interventions were not documented in this study. Availability of adequate equipment for therapy makes it easy for many patients to be seen on time, however, 33% of the participants expressed their concern about the limited number of equipment which resulted in defaulting. This agrees with Chen et al.,^15^ who recorded limited equipment as a system-level barrier to healthcare. Generally, individual-level barriers did not pose challenge to participants' access to physiotherapy except that the expectations of most participants were not met. The majority of the participants reported no challenges with regards to family support, societal attitudes, and comorbidities (hypertension and diabetes) to accessing physiotherapy except one participant who indicated poor family support as a reason for stopping physiotherapy and another who defaulted due to uncontrolled hypertension. In contrast to a study by Nketia-kyere et al.,^24^, attitude of society and other socio-cultural factors were barriers to the health-seeking behaviors of people with neurological conditions. Also, according to Jack et al.,^25^ lack of social or familial support was a barrier to treatment adherence and some of their participants were ready to exercise if accompanied by someone to therapy. This disparity could result from the small sample size for the qualitative component of this study.

Although, about 67% of the participants reported being satisfied with the physiotherapy sessions, 88% of them had stopped treatment. The unmet treatment expectation could account for this observation as the majority of the participants indicated that their expectations for physiotherapy were not met. PD is a neurological condition and it may take some time for treatment outcomes to manifest. However, the majority of PwPD may not have the patience and commitment to physiotherapy to achieve improvement which usually leads to disappointment. Therefore, appropriate education on physiotherapy treatment outcomes is also key to therapy adherence. Knowledge of these barriers is relevant for clinical practice as this provides basis for education for patients/family and PD-specific training for physiotherapists to enhance expertise in managing PwPD. For policymaking, there is the need for more rehabilitation or physiotherapy units to be established in Ghana for easy accessibility. The national health insurance scheme should reimburse cost of physiotherapy without any additional fee. Also, for future research, prospectively assessing the compliance rate and barriers to physiotherapy among a larger sample of PwPD is important to confirm the magnitude of the impact of these challenges on those affected with PD.

The findings of this study have provided information on some of the factors that cause PwPD to stop using physiotherapy services and also serves as a foundation for future research on the topic in Ghana and African as a whole. However, the relatively small study sample and the single center data collection limits the generalizability of the quantitative results and the transferability of the qualitative findings. Also, the retrospective method used in this study limits the scope as the opinions of PwPD who have never used physiotherapy were not captured. The deductive approach to the qualitative data analysis could also introduce some bias though efforts were made to maintain credibility and trustworthiness through self-reflexivity and recognition of the researchers' believes and role^19^.

## Conclusion

The study findings indicated that a notable proportion of PwPD who sought treatment at the PD-clinic of the physiotherapy department in KBTH from May 2013 to December 2021 had failed to comply with their physiotherapy regimen. The primary reasons for this non-compliance were identified as barriers at the system-level. These barriers encompassed various challenges such as difficulties in accessing the clinic due to long distances and associated transportation costs, financial constraints related to treatment expenses, limited duration of therapy sessions, and perceived lack of necessary equipment. Unmet treatment expectation was the key individual-level barrier to physiotherapy.
